# A retrospective study of the clinical diagnosis and treatment of 158 cases of COVID-19

**DOI:** 10.3389/fpubh.2025.1622703

**Published:** 2026-01-08

**Authors:** Xiangqi Chen, Suyun Zhang, Qunying Lin, Xibin Zhuang, Xiangyang Yao, Li Lin, Xiaoyun Chen, Guoxiang Lai, Baosong Xie

**Affiliations:** 1 Department of Pulmonary and Critical Care Medicine, Fujian Medical University Union Hospital, Fuzhou, Fujian, China; 2 Department of Internal Medicine, Fujian Medical University Union Hospital, Fuzhou, Fujian, China; 3 Department of Pulmonary and Critical Care Medicine, Affiliated Hospital of Putian University, Putian, Fujian, China; 4 Department of Pulmonary and Critical Care Medicine, The First Hospital of Quanzhou Afliated to Fujian Medical University, Quanzhou, Fujian, China; 5 Department of Pulmonary Diseases, The First Affiliated Hospital of Xiamen University, Xiamen, Fujian, China; 6 Department of Pulmonary and Critical Care Medicine, Zhangzhou Affiliated Hospital of Fujian Medical University, Zhangzhou, Fujian, China; 7 Department of Pulmonary and Critical Care Medicine, 900th Hospital of the Joint Logistics Support Force, Fuzhou, Fujian, China; 8 Shengli Clinical Medical College of Fujian Medical University, Fuzhou, Fujian, China; 9 Department of Pulmonary and Critical Care Medicine, Fujian Provincial Hospital, Fuzhou, Fujian, China

**Keywords:** Fujian province, COVID-19, clinical characteristics, treatment, severity, risk factors

## Abstract

**Objective:**

This study aimed to summarize the clinical characteristics, management, and outcomes, and to identify independent risk factors associated with the severity in a cohort of coronavirus disease 2019 (COVID-19) patients from early-pandemic Fujian Province, China.

**Methods:**

A total of 158 patients with confirmed COVID-19 were recruited from 10 designed hospitals of the Fujian province between January 22 and February 26, 2020. Their clinical, laboratory, computed tomography imaging, treatment, and outcome data were collected and summarized from electronic medical records. Potential independent risk factors associated with COVID-19 severity were explored by binary logistic multivariate regression.

**Results:**

Of the 158 COVID-19 patients, 36 had mild, 106 had moderate, 8 had severe, and 8 had critical disease severity. The median age was 45 years (interquartile range 35–55) and 81 (51.3%) were men. 8.2% had a history of smoking. 31.6% had chronic underlying conditions, among which hypertension (13.9%), diabetes (7.6%), and liver disease (7.0%) were the most common. The most common initial symptom was fever, followed by cough, sputum expectoration, and muscle soreness. 94.3% of patients had abnormal imaging findings on chest computed tomography or X-ray. Patients with severe/critical disease had significantly more prominent laboratory abnormalities, including an abnormal lymphocyte count and abnormal levels of white blood cells, albumin, aspartate aminotransferase, creatine kinase, lactate dehydrogenase, and D-dimer than mild/moderate patients (all *p* < 0.05). Independent risk factors associated with severe/critical disease included age ≥ 60 years [Odds ratio (OR) = 19.9], WBC ≥ 10 × 10^9^/L (OR = 47.5) and D-dimer > 0.5 mg/L (OR = 5.0) (*p* < 0.05). All patients received antiviral drugs; 126 (79.7%) also received traditional Chinese medicine and 29 (18.4%) received glucocorticoids. As of April 2, 133 patients (84.2%) were cured and discharged, 1 individual died (overall mortality, 0.6%), and the remaining 24 (15.2%) remained hospitalized after data gathering was completed.

**Conclusion:**

COVID-19 patients from Fujian province during early pandemic presented mostly as mild/moderate cases with fever and cough as the initial symptoms. The detection of cellular immune function, coagulation function, myocardial damage, and liver function can help assess the severity of COVID-19. Specifically, the most important risk factors were older age, WBC elevation, and D-dimer elevation. Timely comprehensive treatment might be beneficial in controlling COVID-19.

## Introduction

On December 8, 2019, the first case of pneumonia of unknown etiology was identified in Wuhan, Hubei Province, China (1). On January 7, 2020, through high-throughput sequencing of a throat swab patient sample, the Chinese Center for Disease Control and Prevention identified that the pathogen was a novel coronavirus, originally named 2019-novel coronavirus (2019-nCoV) (2) and later named Severe Acute Respiratory Syndrome Coronavirus 2 (SARS-CoV-2) by the International Committee on Taxonomy of Viruses. The disease arising from SARS-CoV-2 infection was termed 2019 novel coronavirus disease (COVID-19) by the World Health Organization (3). With a rapid transmission (Basic reproductive number, R_0_ = 2.2) and a mortality rate as high as 11%, the COVID-19 epidemic undoubtedly poses a serious threat to global health (4, 5). There have been multiple literature reports on the epidemiological characteristics, clinical manifestations, genomic characteristics, antiviral treatments, and clinical prognosis of COVID-19 (2, 6–10). However, the clinical spectrum of SARS-CoV-2 infection varies in different studies, and COVID-19 can change rapidly and quickly develop into progressive pneumonia, respiratory failure, and even death in some cases (5, 9).

Fujian Province, in southeastern China, presents a notable example of effective outbreak containment. Following the first confirmed case on January 22, 2020, the outbreak was fully controlled by March, with no further local transmission. Despite this success, real-world clinical evidence from China’s southeastern coastal regions remains limited. Most existing reports are constrained by small sample sizes and a narrow range of clinical indicators, failing to adequately capture the disease profile in a naturalistic setting (11, 12).

To address this gap, we conducted a retrospective analysis of confirmed indigenous cases in Fujian Province, admitted to 10 designated hospitals during the COVID-19 early pandemic. The clinical characteristics, laboratory findings, imaging data, diagnosis and treatment scheme, and clinical outcomes of the patients were analyzed. We compared the clinical characteristics of mild/moderate and severe/critical cases of COVID-19, and attempted to identify the independent risk factors for the development of mild/moderate COVID-19 versus severe/critical COVID-19. Through this study, we hope to provide an important reference for the prevention, control, and early clinical diagnosis and treatment of COVID-19.

## Materials and methods

### Clinical data

We recruited laboratory-confirmed indigenous COVID-19 patients admitted to 10 designated hospitals for COVID-19 in Fujian province, China from January 22 to February 26, 2020. The included participants had relatively complete clinical data which were retrospectively collected. The inclusion criteria were: 1) indigenous cases within Fujian Province, and 2) availability of relatively complete clinical data. Cases originating from outside Fujian or those with substantial missing data in their medical records were excluded. A dedicated team of trained physicians and research staff extracted the following data from electronic medical records: clinical characteristics (age, gender, comorbidities, personal histor, past medical history, clinical manifestations, etc.), laboratory findings (complete blood count, liver and renal function tests, coagulation profile, and inflammatory markers, etc.), imaging results, treatment regimens, and clinical outcomes. The patient clinical outcomes (such as discharge, death, length of hospital stay, and virus clearance time) were monitored until the last follow-up date (April 2, 2020). All data were verified by two independent researchers.

### Diagnosis and classification criteria

The diagnosis and classification of COVID-19 were based on the *Diagnosis and Treatment Protocol for Novel Coronavirus Pneumonia (Trial Version 6)* released by the National Health Commission (13). Cases were diagnosed when real-time RT-PCR was positive for SARS-CoV-2 nucleic acid in the respiratory tract or blood samples or the viral gene sequence was highly homologous to known SARS-CoV-2. COVID-19 cases were classified into mild, moderate, severe, or critical as follows. Mild referred to mild clinical symptoms and no sign of pneumonia on imaging. Moderate referred to fever (body temperature > 37.3 °C) and respiratory symptoms with imaging findings of pneumonia. Severe encompassed cases meeting any of the following criteria: (1) respiratory distress (≥ 30 breaths/min), (2) pulse oxygen saturation ≤ 93% at rest, or (3) arterial partial pressure of oxygen (PaO_2_)/fraction of inspired oxygen (FiO_2_) ≤ 300 mmHg (1 mmHg = 0.133 kPa). Critical included cases meeting any of the following criteria: (1) respiratory failure and requiring mechanical ventilation, (2) shock, or (3) concomitant organ failure that necessitated intensive care.

### Research methods

Cases meeting the diagnostic criteria for COVID-19 were clinically classified based on the *Diagnosis and Treatment Protocol for Novel Coronavirus Pneumonia (Trial Version 6)* (13). Mild and moderate cases were grouped into a single “mild/moderate group,” while severe and critical cases were grouped into a “severe/critical group.” The basic data, clinical manifestations, laboratory findings, imaging findings, diagnosis and treatment scheme and efficacy were compared between these two groups, and potential independent risk factors for disease progression were tested.

### Therapeutic regimen

According to the *Diagnosis and Treatment Protocol for Novel Coronavirus Pneumonia (Trial Version 6)* released by the National Health Commission (13), all confirmed cases were treated on isolation wards or negative pressure wards, and appropriate oxygen therapy measures were instituted based on the patient’s respiration, oxygen saturation, and oxygenation index. In addition to routine symptomatic and supportive treatment, antiviral therapy was also utilized. Glucocorticoids, immunoregulation, anti-infection, and adjuvant traditional Chinese medicines were implemented as appropriate following discussion with experts according to the patient’s clinical condition.

### Statistical methods

IBM SPSS 22.0 (Armonk, NY) software was used for data analysis. Measurement data are presented as mean ± standard error (SEM) or median (interquartile range, IQR), as appropriate. Normally-distributed data were compared using Independent-sample Student’s *t*-tests while non-normally distributed data were compared using Mann–Whitney U-tests. Count data are described by [*n* (%)] and compared using the Chi-square and Fisher’s exact tests. Independent risk factors for the development of mild/moderate COVID-19 to severe/critical COVID-19 were screened by binary logistic multivariate regression analysis. *p* < 0.05 was considered to indicate statistical significance.

## Results

### General clinical characteristics

Of 158 COVID-19 patients, the median age was 45 years (interquartile range 35–55) and 81 (51.3%) were men. There were 142 (89.9%) mild/moderate cases and 16 (10.1%) severe/critical cases. 13 (8.2%) had a history of smoking, 8 (5.1%) of drinking alcohol, and 14 (8.9%) of surgery. 31.6% (50/158) had chronic underlying diseases, among which hypertension (22, 13.9%), diabetes (12, 7.6%), and liver disease (11, 7.0%) were the most common comorbidities (Table 1).

**Table 1:** General clinical characteristics of patients infected with 2019-nCoV.

Clinical characteristics	Total (*n* = 158)	Mild/moderate (*n* = 142)	Severe/critical (*n* = 16)	*p* value^a^
Age, median(IQR), y	45 (35–55)	42 (34–52)	72 (60–83)	<0.001
Age ≥ 60 y, *n* (%)	31 (19.6)	19 (13.4)	12 (75.0)	<0.001
Gender				
Male, *n* (%)	81 (51.3)	71 (50)	10 (62.5)	0.343
Female, *n* (%)	77 (48.7)	71 (50)	6 (37.5)	
Smoking history, *n* (%)	13 (8.2)	9 (6.3)	4 (25)	0.036
Alcohol drinking, *n* (%)	8 (5.1)	9 (6.3)	1 (6.3)	1
Operation history, *n* (%)	14 (8.9)	12 (8.5)	2 (12.5)	0.939
Comorbidities, *n* (%)	58 (36.7)	38 (26.8)	12 (75)	<0.001
Hypertension, *n* (%)	22 (13.9)	14 (9.9)	8 (50)	<0.001
Diabetes, *n* (%)	12 (7.6)	8 (5.6)	4 (25)	0.023
Chronic liver disease, *n* (%)	11 (7.0)	10 (7.0)	1 (6.3)	1
Chronic kidney disease, *n* (%)	3 (1.9)	2 (1.4)	1 (6.3)	0.705
Cerebrovascular disease, *n* (%)	3 (1.9)	2 (1.4)	1 (6.3)	0.705
Malignancy, *n* (%)	3 (1.9)	1 (0.1)	2 (12.5)	0.021
Cardiovascular disease, *n* (%)	3 (1.9)	0 (0)	3 (18.8)	<0.001
COPD, *n* (%)	2 (1.3)	1 (0.1)	1 (6.3)	0.483

a *p* values indicate differences between mild/moderate and severe/critical cases.

*p* < 0.05 was considered statistically significant. IQR, Interquartile range; COPD, Chronic obstructive pulmonary disease.

The age, smoking history, and underlying diseases showed a significant difference between the mild/moderate and severe/critical groups. The severe/critical group had higher proportions of older individuals (75% vs. 13.4%, *p* < 0.001), smokers (25% vs. 6.3%, *p* < 0.05), and comorbidities (75% vs. 26.8%, *p* < 0.001) compared to the mild/moderate group (Table 1).

### Common signs and symptoms on admission

The most common initial symptom of 158 patients with COVID-19 was fever (121, 76.6%), followed by cough (113, 71.5%), expectoration (81, 51.3%), and muscle soreness (24, 15.2%). Dizziness, nausea, chest distress, sore throat, anorexia, and diarrhea were rarely seen. The severe/critical group showed a significantly higher proportion of cases with moderate fever (38.1–39.0 °C) than the mild/moderate group (73.3% vs. 37.8%, *p* = 0.009). Among febrile individuals, the median maximum body temperature was higher in the severe/critical group (38.5 °C) than the mild/moderate group (38.0 °C) (*p* = 0.002).

Objective signs of disease were found in 27.4% (37/158) of individuals on admission, the most common of which were pulmonary rales (28, 17.7%). The severe/critical group had a higher proportion of individuals with clinical signs than the mild/moderate group (50.0% vs. 20.4%, *p* = 0.019). In the severe/critical group, 31.3% (5/16) of the cases were admitted to the ICU due to organ failure; this was significantly higher than the 0.1% (1/142) of mild/moderate cases (*p* < 0.05) (Table 2).

**Table 2:** Common signs and symptoms of COVID-19 patients.

Signs and symptoms	Total (*n* = 158)	Mild/moderate (*n* = 142)	Severe/critical (*n* = 16)	*p* value^a^
Fever, *n* (%)	121 (76.6)	106 (76.6)	15 (93.8)	0.162
37.3–38.0°C	66 (54.5)	63 (59.4)	3 (20.0)	0.004
38.1–39.0°C	51 (42.2)	40 (37.8)	11 (73.3)	0.009
>39.0°C	4 (3.3)	3 (2.8)	1 (6.7)	0.995
Highest temperature during hospital admission, median (IQR), °C	38.0 (37.5–38.5)	38.0 (37.4–38.5)	38.5 (38.2–38.7)	0.002
Cough, *n* (%)	113 (71.5)	100 (70.4)	13 (81.3)	0.537
Expectoration, *n* (%)	81 (51.3)	69 (48.6)	12 (75)	0.045
Muscle soreness, *n* (%)	24 (15.2)	21 (14.8)	3 (18.8)	0.959
Dizziness, *n* (%)	21 (13.3)	19 (13.4)	2 (12.5)	1
Nausea, *n* (%)	20 (12.7)	19 (13.4)	1 (6.3)	0.677
Chest distress, *n* (%)	17 (10.8)	14 (9.9)	3 (18.8)	0.508
Sore throat, *n* (%)	14 (8.9)	13 (9.2)	1 (6.3)	1
Anorexia, *n* (%)	14 (8.9)	12 (8.5)	2 (12.5)	0.939
Diarrhea, *n* (%)	14 (8.9)	11 (7.7)	3 (18.8)	0.315
Headache, *n* (%)	13 (8.2)	12 (8.5)	1 (6.3)	1
No symptoms, *n* (%)	8 (5.1)	7 (4.9)	1 (6.3)	1
Obnubilation, *n* (%)	3 (1.9)	0	3 (18.8)	<0.001
Arrhythmia, *n* (%)	5 (3.2)	2 (1.4)	3 (18.8)	0.003
Moist rales, *n* (%)	26 (16.5)	21 (14.8)	5 (31.3)	0.184
Dry rales, *n* (%)	5 (3.2)	4 (2.8)	1 (6.3)	1
Rales, *n* (%)	28 (17.7)	23 (16.2)	5 (31.3)	0.250
No signs, *n* (%)	121 (76.6)	113 (79.6)	8 (50)	0.019
Need ICU, *n* (%)	6 (3.8)	1 (0.1)	5 (31.3)	<0.001

a *p* values indicate differences between mild/moderate and severe/critical patients.

*p* < 0.05 was considered statistically significant. IQR, Interquartile range; ICU, Intensive care unit.

### Laboratory and imaging findings on admission

The severe/critical group had decreased lymphocyte counts, lymphocyte percentages, red blood cell counts, and albumin (ALB) levels, and increased levels of aspartate aminotransferase (AST), creatine kinase (CK), lactate dehydrogenase (LDH), D-dimer, and C-reactive protein (CRP) compared with the mild/moderate group (all *p* < 0.05). The proportion of patients with an increased white blood cell count (WBC) was significantly higher in the severe/critical than the mild/moderate group. Procalcitonin (PCT) levels rose markedly in the severe/cricitial group, but the difference was not statistically significant between the two groups.

The most common abnormal manifestation evident in the first chest imaging examination (chest CT or X-ray) was pneumonia (149, 94.3%), followed by multiple patchy shadows or ground glass opacity (100, 63.3%). In the whole cohort, bilateral lung involvement occurred in 74.7% (118/158) of patients, 19.6% (31/158) showed unilateral pneumonia, and 5.7% (9/158) had no imaging changes. In the severe/critical group, all showed chest imaging manifestations; 93.8% of cases had bilateral lung involvement and 6.2% had unilateral involvement. The mild/moderate group, showed bilateral (72.5%), unilateral (21.1%), or no (6.3%) imaging changes. As such, there were no significant differences between these groups in imaging manifestations and lung lobe involvement (all *p* > 0.05) (Table 3).

**Table 3:** Laboratory and imaging findings of COVID-19 patients on admission to hospital.

Laboratory and imaging findings	Total (*n* = 158)	Mild/moderate (*n* = 142)	Severe/critical (*n* = 16)	*p* value^a^
White blood cell count (×10^9^/L)	5.7 ± 2.4	5.6 ± 1.9	6.8 ± 5.1	0.350
<4 (*n*, %)	36 (22.8)	32 (22.5)	4 (25.0)	1
4–10 (*n*, %)	116 (73.4)	107 (75.4)	9 (56.2)	0.180
>10 (*n*, %)	6 (3.8)	3 (2.1)	3 (18.8)	0.009
Neutrophil count (×10^9^/L)	4.7 ± 3.4	4.2 ± 6.2	5.3 ± 4.9	0.519
Lymphocyte count (×10^9^/L)	1.4 ± 0.8	1.4 ± 0.8	1.2 ± 0.7	0.055
≤1 (*n*, %)	45 (28.5)	36 (25.4)	9 (56.3)	0.021
>1 (*n*, %)	113 (71.5)	106 (74.6)	7 (43.7)	
Lymphocyte percentage (%)	26.4 ± 11.0	26.9 ± 10.6	22.1 ± 13.9	0.049
Erythrocyte count (×10^9^/L)	4.5 ± 0.7	4.6 ± 0.7	4.1 ± 0.9	0.032
Hemoglobin (g/L)	137.5 ± 25.2	137.2 ± 23.8	122.7 ± 31.9	0.052
Platelet count (×10^9^/L)	206.8 ± 73.7	208.2 ± 72.8	194.4 ± 82.6	0.479
ALB (g/L)	40.2 ± 4.8	40.6 ± 4.3	36.2 ± 6.3	0.007
Globulin (g/L)	29.6 ± 4.5	29.4 ± 4.2	30.9 ± 6.2	0.559
Alanine aminotransferase (U/L)	33.1 ± 30.9	32.8 ± 31.6	35.4 ± 23.7	0.753
≤40 (*n*, %)	122 (77.2)	111 (78.2)	11 (68.8)	0.591
>40 (*n*, %)	36 (22.8)	31 (21.8)	5 (31.2)	
Aspartate aminotransferase (U/L)	29.1 ± 15.5	27.6 ± 15.0	42.8 ± 14.0	<0.001
AST/ALT >1 (*n*, %)	78 (49.4)	65 (45.8)	13 (81.3)	0.009
Creatine kinase (U/L)	117.9 ± 142.6	100.6 ± 97.7	270.1 ± 318.1	0.045
≤190 (*n*, %)	136 (86.1)	127 (89.4)	9 (56.3)	0.001
>190 (*n*, %)	22 (13.9)	15 (10.6)	7 (43.7)	
Lactate dehydrogenase (U/L)	306.8 ± 184.7	289.9 ± 166.4	456.5 ± 265.1	0.001
≤245 (*n*, %)	80 (50.6)	78 (54.9)	2 (12.5)	0.001
>245 (*n*, %)	78 (49.4)	64 (45.1)	14 (87.5)	
D-dimer >0.5 mg/L (*n*, %)	40 (25.3)	32 (22.5)	8 (50)	0.036
C-reactive protein (mg/L)	29.2 ± 39.1	18.9 ± 21.1	93.5 ± 6.8	0.000
Procalcitonin (ng/mL)	0.5 ± 2.3	0.4 ± 2.3	1.1 ± 2.4	0.257
Procalcitonin > 0.5 ng/mL (*n*, %)	59 (37.3)	55 (38.7)	4 (25.0)	0.421
Chest imaging findings at illness onset, (*n*, %)				
Pneumonia	149 (94.3)	133 (93.7)	16 (100)	0.640
Patchy shadows or ground glass opacity	100 (63.3)	90 (63.4)	10 (62.5)	1
Unilateral distribution	31 (19.6)	30 (21.1)	1 (6.2)	0.233
Bilateral distribution	118 (74.7)	103 (72.5)	15 (93.8)	
Normal	9 (5.7)	9 (6.3)	0	0.640

a *p* values indicate differences between mild/moderate and severe/critical patients.

*p* < 0.05 was considered statistically significant. AST, Aspartate aminotransferase; ALT, Alanine aminotransferase.

### Therapeutic measures and clinical outcomes

Antiviral treatment with 1–5 antiviral drugs (including α-interferon, lopinavir/ritonavir, ribavirin, arbidol, oseltamivir, and chloroquine phosphate) was administered to all of the 158 patients. Of these, 67.7% (107/158) received two or more antiviral drugs. There was no significant difference in the therapeutic regimen between groups (*p* = 0.182).

In the severe/critical group, glucocorticoids and immune enhancers (i.e., gamma globulin/thymalfasin for injection) were administered to significantly more patients than in the mild/moderate group (56.3% vs. 14.1%, *p* < 0001; 68.8% vs. 30.3%, *p* = 0.005). The proportion of patients receiving oxygen therapy was also significantly higher in the severe/critical group (87.5% vs. 36.6%, *p* < 0.00).

The most common complications of COVID-19 infection included liver injury (39, 24.7%), gastrointestinal tract injury (32, 20.3%), and respiratory failure (9, 5.7%). The incidence rate of one of the above complications was higher in the severe/critical group compared to the mild/moderate group.

As of April 2, 2020, of the 158 cases of COVID-19, 133 (84.2%) were cured and discharged, 24 (15.2%) had improved symptoms but remained hospitalized, and 1 (0.6%) had died. In the severe/critical group, the median length of hospital stay and days to nucleic acid negative conversion were significantly longer than in the moderate group (all *p* < 0.05) (Table 4).

**Table 4:** Treatments and prognosis of patients with COVID-19.

Treatments and prognosis	Total (*n* = 158)	Mild/moderate (*n* = 142)	Severe/critical (*n* = 16)	*p* value^a^
Treatment				
Antiviral drugs, mean (SEM)	2.6 ± 1.0	2.7 ± 1.0	2.3 ± 0.9	0.182
Single, *n* (%)	51 (32.3)	44 (31.0)	7 (43.7)	0.301
Multiple, *n* (%)	107 (67.7)	98 (69.0)	9 (56.3)	
Glucocorticoid therapy, *n* (%)	29 (18.4)	20 (14.1)	9 (56.3)	<0.001
Administration of immune enhancers (gamma globulin/thymalfasin for injection), *n* (%)	54 (34.2)	43 (30.3)	11 (68.8)	0.005
Chinese medicine, *n* (%)	126 (79.7)	114 (80.3)	12 (75)	0.805
Oxygen therapy, *n* (%)	66 (41.8)	52 (36.6)	14 (87.5)	<0.001
Complications, *n* (%)	39 (24.7)	26 (18.3)	13 (81.3)	<0.001
Acute liver injury	39 (24.7)	29 (20.4)	10 (62.5)	0.001
Adverse drug reaction	32 (20.3)	25 (17.6)	7 (43.8)	0.032
Respiratory failure	9 (5.7)	2 (1.4)	7 (43.8)	<0.001
Prognosis, *n* (%)				
Discharge	133 (84.2)	126 (88.7)	7 (43.8)	<0.001
Hospitalisation	24 (15.2)	16 (11.3)	8 (50)	
Death	1 (0.6)	0	1 (6.3)	
Hospitalized days	20.2 ± 8.1	19.6 ± 8.0	24.8 ± 7.2	0.006
Virus clearance days	13.1 ± 9.0	12.4 ± 8.9	19.2 ± 7.2	0.002

a *p* values indicate differences between mild/moderate and severe/critical patients.

*p* < 0.05 was considered statistically significant. IQR, Interquartile range; SEM, Standard error.

**Figure 1:**
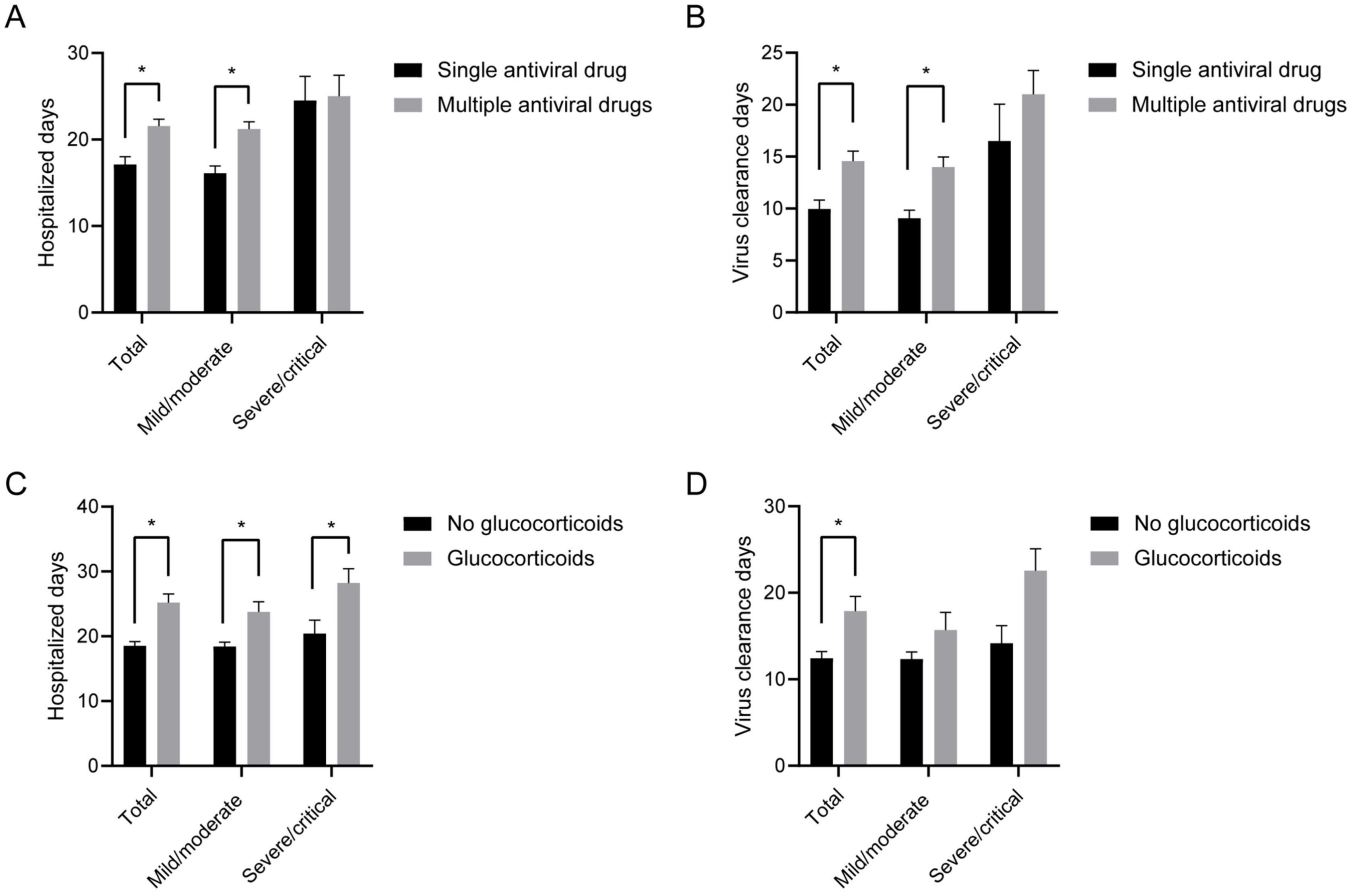
Statistical chart of prognosis based on the types of antiviral drugs or use of glucocorticoids. Compared with a single antiviral drug, the combination of multiple antiviral drug was associated with a longer length of hospital stay **(A)** and days to nucleic acid negative conversion **(B)**. The administration of glucocorticoids was associated with a longer length of hospital stay **(C)** and days to nucleic acid negative conversion **(D)**. * *p* < 0.05.

Based on these data, we explored the effects of administration of single vs. multiple antiviral drugs and the incorporation of glucocorticoid therapy on the prognosis of patients with COVID-19. Since only 1 patients died by the last follow-up visit (an overall survival rate of 99.4%), the prognosis was assessed based on the length of hospital stay and days of virus clearance, rather than by testing against survival vs. mortality. As shown in Figure 1, in our observational analysis, the use of multiple antiviral drugs (compared to a single drug) or low-dose glucocorticoids (compared to no glucocorticoids) was not significantly associated with a reduction in the length of hospital stay or virus clearance time.

### Independent risk factors for the development of severe/critical COVID-19

As presented in the above results, age, chronic underlying diseases, WBC, lymphocytes, LDH, and D-dimer were independently associated with disease severity. Then we conducted binary logistic multivariate regression analyses with severe or critical COVID-19 as the dependent variable against the above single predictor variables. We found that age ≥ 60 years, WBC ≥ 10 × 10^9^/L and D-dimer >0.5 mg/L were independent risk factors for the severity of COVID-19 (Table 5).

**Table 5:** Logistic regression analysis of risk factors associated with the severity of COVID-19.

Characteristics and findings	Wald *x^2^*	OR	95% CI	*p* value
Age (≥60 ys vs. <60 ys)	11.974	19.914	3.659–108.392	0.001
Comorbidities (Yes vs. No)	4.061	5.702	1.049–30.991	0.044
White blood cell count (≥10 × 10^9^/L vs. <10 × 10^9^/L)	6.469	47.497	2.424–930.542	0.011
Lymphocyte count (≤1.0 × 10^9^/L vs. >1.0 × 10^9^/L)	2.717	3.581	0.786–16.316	0.099
Lactate dehydrogenase (>245 U/L vs. ≤245 U/L)	3.030	5.021	0.816–30.894	0.082
D-dimer (>0.5 mg/L vs. ≤0.5 mg/L)	4.667	6.012	1.181–30.608	0.031

## Discussion

In this study, we retrospectively analyzed data from 158 COVID-19 patients admitted to 10 designated hospitals for COVID-19 in Fujian Province, China, from January 10 to February 26, 2020. 10.1% of them were severe/critical patients, lower than the reported 17.7% in Wuhan but higher than the 7.0% in areas outside of Wuhan severe/critical group was 6.3%, which was lower than that reported in multiple case series in Wuhan [e.g., Huang et al. (15%) (9) and reported by Chen *et al*. (11%) (14)]. The proportion and mortality rate of severe/critical cases in Fujian Province were lower than in Hubei Province, which may be attributed to proactive preventive measures, better preparation of medical resources, better-trained disease identification and detection capabilities, early detection of asymptomatic and mild cases, effective supportive treatment in designated hospitals, and education of patients.

The median age of this cohort was 45 years and severe/critical cases were found to be older than mild/moderate cases. The results of binary logistic regression analysis further revealed that being aged ≥ 60 years was a possible independent risk factor for higher disease severity, which may be related to weakened immunity and the presence of comorbidities in the older population (15–17). In 2020, Chen et al. (4) reported that men are more susceptible to COVID-19, however that study enrolled workers of the Huanan Seafood Market in Wuhan, which is predominantly male. In this study, the proportion of men was comparable to that of women. There was a trend for a greater proportion of men in the severe/critical group but this was not statistically significant. There is no compelling evidence that supports that men are at greater risk of developing COVID-19 or displaying a more severe clinical phenotype. We found that, compared to the mild/moderate group, the severe/critical group had a greater proportion of patients with a smoking history and underlying diseases such as diabetes, hypertension, and coronary heart disease. This corresponds with the findings of Zheng et al. (18). Our regression analysis showed that complicated chronic underlying disease presented an independent risk factor for severe/critical COVID-19. One possible reason is that the harmful substances in cigarettes impair the lung function of smokers, reducing their resistance to viral pathogens and increasing the risk of developing an acute respiratory distress syndrome (ARDS). In the presence of underlying diseases such as diabetes, hypertension, and coronary heart disease, patients are in a long-term stress state, the vascular structure is damaged, and immunity is weakened, so they are more prone to severe or critical COVID-19 when infected with SARS-CoV-2 (18). Notably, the prevalence of smoking in our cohort (8.2%, 13/158) was notably lower than the national average in China (19). This underrepresentation of smokers among hospitalized COVID-19 patients has been observed elsewhere and is often referred to as the ‘smoker’s paradox’ (20). The underlying mechanisms remain unclear but may involve immunomodulatory effects of nicotine or cross-reactive immunity from prior exposures (21, 22). Nonetheless, it is crucial to emphasize that among patients who are hospitalized, existing evidence, consistent with our findings, suggests that smokers often experience a more severe disease course.

The typical initial symptoms of COVID-19 patients from Fujian Province included fever, accompanied by cough, expectoration, and muscle soreness. Less commonly they experienced nausea, anorexia, and diarrhea. In 2020, Wang et al. (8) analyzed the clinical characteristics of 138 hospitalized COVID-19 patients in Wuhan, China, and found that the most common initial symptom was fever, followed by cough and muscle soreness; gastrointestinal symptoms such as diarrhea, nausea, and vomiting were rarely present. In contrast, Zhou and Yang et al. reported in 2021 (23, 24) that in their cohort gastrointestinal symptoms such as anorexia, diarrhea, nausea, and vomiting were the first manifestations of COVID-19. The results of our study parallel those of the former. It is important to note that the clinical symptoms of COVID-19 may change with the emergence of new variants and there will need to be an ongoing effort to analyze emerging cases in future studies.

Patients with COVID-19 show diverse clinical and laboratory manifestations of their disease. We found that several laboratory indicators differed between the groups. WBCs are normal or reduced in the early stagse of COVID-19 (9), and elevation of WBC often indicates secondary bacterial infection, persistent inflammation, or disease progression (25). Relevant studies have shown that there is a close association between WBC and the prognosis of patients with COVID-19. For example, Huang et al. (9) found that a WBC < 4 × 10^9^/L indicates a better clinical prognosis, and Moradi et al. (25) argued that elevation of WBC is an independent risk factor for death in critically ill patients. In this study, WBC was higher in severe/critical cases than in mild/moderate cases, and a WBC ≥ 10 × 10^9^/L was an independent risk factor for disease progression. Despite the notably wide confidence interval for the WBC count, which likely reflects the limited number of patients with leukocytosis in the severe or critical subgroup, a strong correlation with disease severity was observed. This finding underscores the clinical importance of an elevated WBC count as a significant marker, although the exact magnitude of its effect should be interpreted with caution. Therefore, early detection of WBC can provide some reference for disease evaluation, diagnosis, and treatment. Severe/critical cases had a significantly increased level of CRP, and significantly decreased percentages and absolute values of lymphocytes than mild/moderate cases. These indicators suggest that the mechanism of occurrence and development of COVID-19 may be similar to that of pneumonia caused by SARS and MERS coronaviruses, and that there may be a process of cellular immunity impairment (8, 26, 27). D-dimer, a product of fibrin degradation, can serve as a common indicator for evaluating coagulation function. D-dimer can be increased during SARS-CoV-2 infection, and the resultant venous thrombosis or pulmonary embolism will complicate the condition (28–30). In this study, D-dimer was elevated in some patients, especially in severe/critical cases, similar to the results of previous studies. Elevation of D-dimer was further confirmed to be an independent risk factor for the development of severe/critical COVID-19, suggesting that SARS-CoV-2 infection affects the coagulation system. Quantification of plasma D-dimer levels may provide clinicians with a tool that helps the early identification of patients prone to disease progression, thereby facilitating early therapeutic responses. The levels of LDH, CK, and AST were also significantly higher in severe/critical cases than in mild/moderate cases. This also suggests that SARS-CoV-2 may damage myocardial cells directly or indirectly through a cellular inflammatory storm (31, 32). The decrease in ALB was especially evident in severe/critical cases; we speculate that this may be related to the impairment of ALB synthesis in the liver resulting from viral damage to hepatocytes, and the enhanced consumption of ALB due to inflammation and fever (33–35). In short, infection with SARS-CoV-2 negatively impacts multiple organs and systems and causes widespread damage to the cellular immune system, coagulation system, heart, and liver. However, the specific damage mechanisms need further elucidation.

In this study, treatments including antiviral drugs, oxygen therapy, immunomodulatory drugs, and adjuvant traditional Chinese medicine were analyzed. As of April 2, 2020, 157 patients were cured or clinically improved, and there were only 1 deaths. Due to the rapid progression of COVID-19 and the lack of specific antiviral drugs, combination antiviral drugs are commonly used. Considering the potential adverse effects of these drugs, however, combinations of three or more antiviral drugs were not recommended in the *Diagnosis and Treatment Protocol for Novel Coronavirus Pneumonia (Trial Version 8)*. To date, the efficacy of any single antiviral drug has not been fully assessed. Some experts believe that two or more effective antiviral drugs may be combined to reduce virus replication and shorten its clearance time, but the use of multiple antiviral drugs in patients at different stages of COVID-19 remains controversial (36–39). In this retrospective study, the effects of a single or multiple antiviral drug(s) on the length of hospital stay and days to nucleic acid negative conversion were preliminarily explored. We found that a combination of multiple antiviral drugs was not associated with a better prognosis as compared to a single antiviral drug among COVID-19 patients (for total, or mild/moderate, or severe/critical cases). As recommended by the *Diagnosis and Treatment Protocol for Novel Coronavirus Pneumonia (Trial Version 8)*, patients with rapid disease progression and excessive activation of the inflammatory response can be given short-term low-dose glucocorticoids as appropriate for 3–5 d (40). However, the treatment of COVID-19 with glucocorticoids has not been extensively studied, and its effectiveness remains controversial due to its strong immunosuppressive function and adverse effects. According to a recent large-scale randomized clinical trial, dexamethasone can reduce the 28-day mortality rate in critical COVID-19 patients undergoing invasive mechanical ventilation or oxygen therapy, but it does not benefit patients who are not receiving respiratory support (41). Wu et al. (42) retrospectively analyzed the treatment of 201 patients with COVID-19 and found that the mortality rate of patients suffering from ARDS can be reduced by glucocorticoid intervention. In contrast, in a meta-analysis of 12 observational studies and 1 randomized controlled trial involving 16,977 patients, Haytham Tlayjeh et al. (43) found that the use of glucocorticoids was not associated with reduced short-term mortality in hospitalized patients with COVID-19 of varying severity. In a retrospective analysis involving 244 patients with COVID-19, Lu et al. (44) reported that glucocorticoids do not alter the chances of survival of critical COVID-19 patients. Moreover, it was pointed out in a retrospective study that glucocorticoid intervention does not benefit COVID-19, but delayed virus clearance in 2 patients who had concomitant HBV infection (45). In this study, some patients were treated with glucocorticoid therapy to suppress cytokine storms and relieve pulmonary exudation. Based on the risk of severe adverse reactions associated with high-dose glucocorticoids in SARS-CoV-1 (46) and the lack of improved survival in COVID-19 (47, 48), low-dose glucocorticoids were administered to only 18.4% of SARS-CoV-2-infected patients with severe pulmonary exudation in our study. However, we found that virus clearance time and length of hospital stay were significantly longer among patients using glucocorticoids, consistent with the statement in the *Diagnosis and Treatment Protocol for Novel Coronavirus Pneumonia (Trial Version 8)*. The possible reason is that glucocorticoids inhibit the host immune response and pathogen elimination while suppressing the body’s inflammatory response. As a result, the time to nucleic acid negative conversion is prolonged, thus extending the length of hospital stay. We acknowledge the inherent limitations of our treatment efficacy analyses, which are exploratory and observational. In the absence of adjustment for potential confounders, these results cannot establish definitive conclusions on therapeutic efficacy and should be interpreted with caution. More high-level evidence is needed to assess the effects of these therapeutic strategies on clinical improvement and prognosis of COVID-19 patients.

In this paper, we analyzed the diagnosis and treatment of patients with COVID-19 and the main clinical characteristics, principles of diagnosis and treatment, clinical outcomes, and independent risk factors for development of severe/critical diseases were preliminarily determined. The cohort was characterized by mostly mild/moderate cases, with initial symptoms including fever and cough. The severe/critical patients tended to be older, were more likely to smoke and have underlying medical conditions. The severe/critical cases also had greater changes in laboratory indicators such as WBC, lymphocytes, ALB, AST, CK, LDH, and D-dimer. Age ≥ 60 years, WBC ≥ 10 × 10^9^/L, and D-dimer > 0.5 mg/L were determined to be independent risk factors for the development of severe/critical COVID-19, and may serve as useful clinical indicators to assist clinicians predict emergent adverse clinical states and to guide clinical decision-making. Active comprehensive treatments including antiviral drugs, oxygen therapy, immunoregulation, and traditional Chinese medicine are expected to achieve good effects. However, combination antiviral drugs (vs. a single antiviral drug) or low-dose glucocorticoids (vs. no glucocorticoids) were not significantly beneficial. The efficacy of therapeutic strategies for COVID-19 patients requires more high-level evidence.

Our study has several limitations that should be considered. First, as a retrospective study with a relatively small number of severe/critical cases, it carries an inherent risk of selection bias. Second, the data were collected from 10 different hospitals, where variations in reference ranges for some laboratory indicators may have affected the precision of our analysis. Third, our analysis of treatment outcomes did not adjust for all potential confounders, which limits causal inference regarding the observed associations. Furthermore, the generalizability of our findings may be constrained by the low overall mortality rate in our cohort when considering populations with higher mortality rates. Additionally, we lacked detailed data on key clinical variables, such as the time from symptom onset to hospital admission, which is crucial for a nuanced interpretation of disease progression and treatment timing. Finally, the absence of data on psychological support interventions represents another gap that warrants attention in future research.

## Data Availability

The original contributions presented in the study are included in the article/supplementary material, further inquiries can be directed to the corresponding authors.
